# Acceptance‐Hesitancy of COVID‐19 Vaccination and Factors Affecting It in Adults: Systematic Review Study

**DOI:** 10.1002/iid3.70076

**Published:** 2024-11-21

**Authors:** Atieh Darbandi, Maryam Koupaei, Parisa kiani, Roya Ghanavati, Parisa Najafi, Jalil Hosseini, Mohammad Reza Shokouhamiri, Arezoo Asadi, Roxana Parsapour

**Affiliations:** ^1^ Molecular Microbiology Research Center Shahed University Tehran Iran; ^2^ Department of Microbiology and Immunology, School of Medicine Kashan University of Medical Sciences Kashan Iran; ^3^ Department of Bacteriology, Faculty of Medical Sciences Tarbiat Modares University Tehran Iran; ^4^ School of Medicine Behbahan Faculty of Medical Sciences Behbahan Iran; ^5^ Faculty of Sports and Exercise Science University Malaya Kuala Lumpur Malaysia; ^6^ Men's Health & Reproductive Health Research Centre, Shohada Hospital Tajrish Shahid Beheshti University of Medical Sciences Tehran Iran; ^7^ Department of Mycology and Parasitology, Faculty of Medical Sciences Golestan University of Medical Sciences Gorgan Iran; ^8^ Endocrine Research Center, Institute of Endocrinology and Metabolism Iran University of Medical Sciences Tehran Iran

**Keywords:** COVID‐19, cross‐sectional studies, vaccination hesitancy, vaccination refusal, vaccine acceptance

## Abstract

**Background:**

Despite the advent of vaccines against COVID‐19, there is considerable variation in the acceptance and hesitancy towards the vaccination program across different countries. The objective of this study was to ascertain the prevalence of hesitancy and acceptance regarding the use of the vaccine against the novel coronavirus, also known as COVID‐19, and to identify the factors that influence these attitudes.

**Materials and Methods:**

All the cross‐sectional studies were retrieved from the PubMed databases, the Web of Science ISI, Scopus, and the Cochrane Library. Papers published in English between 2 November 2019 and 23 May 2023 were subjected to further assessment based on their title, abstract, and main text, with a view to ensuring their relevance to the present study.

**Results:**

Following an exhaustive investigation, 59 studies were selected for screening in this systematic review. The most frequently employed method of data collection was the online survey. The study sample comprised 59.12% women and 40.88% men, with ages ranging from 16 to 78 years. The proportion of individuals accepting the vaccine ranged from 13% to 96%, while the proportion of those exhibiting hesitancy ranged from 0% to 57.5%. The primary reasons for accepting the COIVD‐19 vaccine were a heightened perception of risk associated with the virus and a general trust in the healthcare system. The most frequently cited reasons for vaccine hesitancy in the context of the ongoing pandemic include concerns about the potential dangers of the vaccines, the rapid pace of their development, the possibility of adverse effects (such as infertility or death), and the assumption that they have been designed to inject microchips.

**Discussion:**

A variety of socio‐demographic factors are implicated in determining the rate of vaccine acceptance. A number of socio‐demographic factors have been identified as influencing vaccine acceptance. These include high income, male gender, older age, marriage, the presence of older children who have been vaccinated and do not have chronic diseases, high education, and health insurance coverage.

**Conclusion:**

Eliminating vaccine hesitancy or increasing vaccine acceptance is a crucial factor that should be addressed through various means and in collaboration with regulatory and healthcare organizations.

## Graphical abstract

SARS‐CoV‐2 poses one of the most significant challenges in today's world. Vaccine acceptance is influenced by factors such as age, gender, and various socioeconomic variables. Various socio‐demographic factors are involved in determining the rate of vaccine acceptance.

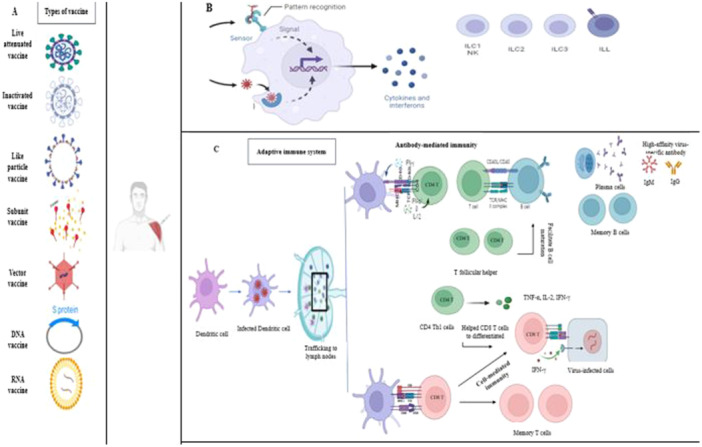

**Abbreviations**
CDC: Centers for Disease Control and Prevention
CHWs: community health workers
COVID‐19: coronavirus disease 2019
FDA: Food and Drug Administration
HCWs: healthcare workers
LMIC: low‐income and middle‐income countries
PRISMA: preferred reporting items for systematic reviews and meta‐analyses
SAGE: strategic advisory group of experts
SARS‐CoV‐2: severe acute respiratory syndrome coronavirus 2
UAE: United Arab Emirates
UK: United Kingdon
USA: United States of America
WHO: World Health Organization

## 1 Introduction

A virulent and contagious virus, designated as the coronavirus, commenced its global dissemination and was classified as a pandemic by the World Health Organization (WHO) in 2020 [1]. The declaration of the global emergency associated with coronavirus disease 2019 (COVID‐19) has persisted for over 3 years. While the WHO has proclaimed the conclusion of the pandemic emergency associated with the virus in May 2023, it has also underscored that the virus continues to represent a significant global health concern [2]. Nevertheless, the situation remains fraught with numerous concerns and challenges. Since the emergence of the novel severe acute respiratory syndrome coronavirus 2 (SARS‐CoV‐2) in December 2019, there have been over 766 million documented cases and 6.9 million deaths worldwide as of 24 May 2023. In such a situation, vaccination and achieving herd immunity are considered the most effective measures to control the spread of infection and improve the health status of the population [1]. Nevertheless, despite the availability of vaccines against the SARS‐CoV‐2 virus, achieving 100% vaccination rates in any population has not been realized. There is considerable variability in vaccine acceptance among different countries, despite the invention of vaccines against the virus [2, 3, 4]. Vaccine hesitancy, as defined by the WHO, refers to the delay in accepting or refusing vaccination despite its availability [1]. A number of studies have indicated that vaccine hesitancy often stems from concerns regarding the safety of the vaccines in question, particularly in regard to potential long‐term side effects. Among the most common reasons for hesitancy in relation to the vaccine for the novel coronavirus, SARS‐CoV‐2, are fears that the vaccines are dangerous, that they have been developed too quickly, that they cause adverse effects (such as infertility or death), or that they have been designed to inject microchips. The WHO has identified vaccine hesitancy as one of the top ten threats to global health [5].

**Figure 1:**
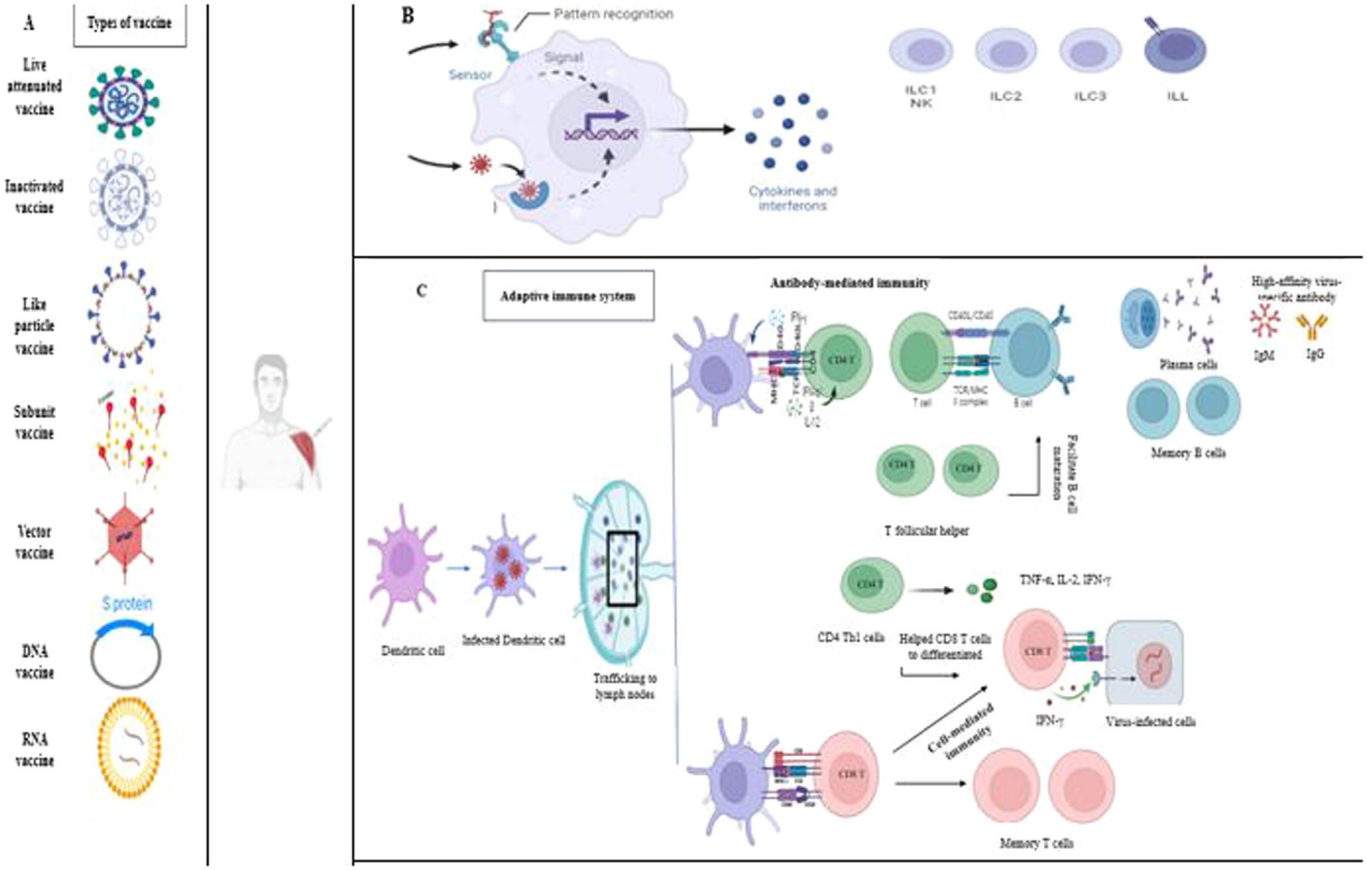
Summary of adaptive antiviral immune responses, (A) Number of potential vaccines entered into clinical trials, (B) When the vaccine is administered, it can be detected by internal sensors within cells, triggering the activation of the innate immune system, which leads to the creation of cytokines and IFNs, (C) adaptive immunity system: Dendritic cells mature in response to the interferons (IFNs) and cytokines produced, and then travel to peripheral lymphoid organs. In these organs, mature dendritic cells present antigens to T cells using either major histocompatibility complex (MHC) class I or MHC class II complexes, which bind to CD8+ or CD4+ T cells respectively. Through MHC‐II, dendritic cells activate CD4+ T cells or helper T cells and regulate the immune response by secreting cytokines. They also activate B cells, initiating antibody class switching via CD40/CD40L signaling. Activated B cells transform into plasma cells that produce various types of antibodies, crucial for the adaptive immune response. Additionally, dendritic cells promote the expansion and differentiation of CD8+ T cells by producing interleukin‐2 (IL‐2) and expressing interferon‐gamma (IFN‐γ), tumor necrosis factor‐alpha (TNF‐α), and IL‐2. These CD8+ T cells can mature into cytotoxic T lymphocytes (CTLs) upon activation, generating perforin and other cytokines that can enhance vaccine effectiveness. Circulating T follicular helper (cTfh) cells aid in B‐cell maturation and the production of high‐affinity antibodies. Image created by PM using BioRender (https://biorender.com/).

Side effects are considered as any adverse medical event after immunization. When the vaccine enters the body, the body's innate and acquired immune responses are activated and many pro‐inflammatory cytokines and interleukins are produced. Figure 1 shows the body's immune reactions after exposure to an antigen either from a pathogen or a vaccination.

Vaccine hesitancy represents a significant global challenge, shaped by a multitude of intricate factors [6]. These encompass the timing and geographical context of vaccination, the specific vaccine in question, the intended audience, and a range of psychological, cognitive, and demographic elements [7]. The term “vaccine acceptance” is defined as “the degree to which individuals accept, question, or refuse vaccination.” It is of great consequence in determining the rate of uptake of vaccines and the overall success of vaccine distribution [8]. Some motivating factors that can minimize the burden of vaccine hesitancy at the population, governmental, and worldwide levels are structural (i.e., government, country), extrinsic (i.e., family, friends), intrinsic (i.e., self‐perception), and other factors (financial and nonfinancial). Protection Motivation Theory states that elements like vaccination views, efficacy, the severity of health threats, and a low incidence of community illnesses can affect a person's motivation to get vaccinated, making them crucial components of engaging in healthy behavior. Particularly, worries about the risks or side effects, as well as social and peer pressure, can significantly affect a person's willingness to get vaccinated [9, 10]. The primary motivation for individuals to receive the vaccine is to gain immunity against the disease, followed by their confidence in the safety of the vaccine. Studies have demonstrated that trust in the healthcare system and an elevated perception of risk associated with SARS‐CoV‐2 are crucial factors in promoting vaccine acceptance.

The objective of this study was to ascertain the prevalence of hesitancy and acceptance regarding the SARS‐CoV‐2 vaccine and to identify the factors that influence these attitudes. The findings of this study can inform decision‐makers and stakeholders engaged in the field of communicable disease prevention, particularly with regard to the optimization of vaccination strategies, the enhancement of the coverage of immunization programmed against infectious diseases, and the implementation of community‐based interventions in this domain.

## 2 Materials and Methods

This study was conducted following the guidelines of the Preferred Reporting Items for Systematic Reviews and Meta‐Analyses (PRISMA) study protocol registered on PROSPERO: CRD42023443404 (https://www.crd.york.ac.uk/prospero/display_record.php?RecordID=443404) [11]. The search strategy was employed using the keywords “Vaccination Acceptance,” “Vaccine Acceptance,” “COVID‐19,” “Adult,” “Vaccination Hesitancy,” “Vaccine Hesitancy,” “Vaccine Refusal,” and “Vaccination Refusal” on PubMed databases, Web of Science ISI, Scopus, Cochrane, from November 2, 2019, to May 23, 2023.

### 2.1 Record Screening and Eligibility

Research was conducted by two independent researchers (PN, and JH), and papers indexed in two or more databases were considered only once. References list of all the related articles were investigated to identify any ignored articles. A third researcher (AD) reviewed the results to ensure that all eligible articles were evaluated. The relevance of the papers was assessed based on their title, and abstract, followed by a detailed review of the studies to determine their suitability based on specific eligibility criteria. The studies were meticulously chosen by the following criteria: Population (P), Intervention (I), Comparator (C), and Outcomes (O), commonly referred to as PICO.

**Populations**. Articles that included people who had delayed acceptance or refusal of COVID‐19 vaccines despite their availability. No additional restrictions on population are considered.

**Intervention/Phenomenon of Interest.** The systematic review focused on research policies, barriers, and enablers for COVID‐19 vaccination coverage, performance, and productivity.

**Comparison.** No criteria for comparison were applicable.

**Outcomes.** Any reported impact on vaccine hesitancy and acceptance.

The inclusion criteria for considering full‐text publications were as follows: (i) Sources published between the years 2019–2023, when the WHO declared the nonemergency status of COVID‐19 as a pandemic threat; (ii) Types of quantitative and qualitative studies. The exclusion criteria were as follows: (i) Animal experiments; (ii) Congress papers; (iii) Reviews, meta‐analyses, case reports, letters to the editor, and correspondence; (iv) Clinical feature summaries; (v) non‐English papers; and (vi) Studies with insufficient information.

### 2.2 Data Extraction

Independent researchers carried out data extraction (PN, and MS). Any discrepancies that arose were resolved through consensus, and if an agreement could not be reached, another researcher was consulted (AA). The variables that were extracted included: Education, Acceptance Rate (%), Hesitancy Rate, Refusal Rate, Pregnancy, Dose, Type of vaccine, Participants, Sex, Mean Age (±SD) of Participants, Characteristics of Participants, Study Design, Survey Modality, and Country.

**Quality assessment:** The quality of the references was assessed using the Joanna Briggs Institute (JBI; The Joanna Briggs Institute, 2014) [12]. The papers with high quality were included in the current study.

### 2.3 Analysis

This review provides a descriptive summary of the included empirical cross‐sectional studies. We refrained from any statistical combination of the results from the different studies because of the differences in their design.

## 3 Results

### 3.1 Search Results

**Figure 2:**
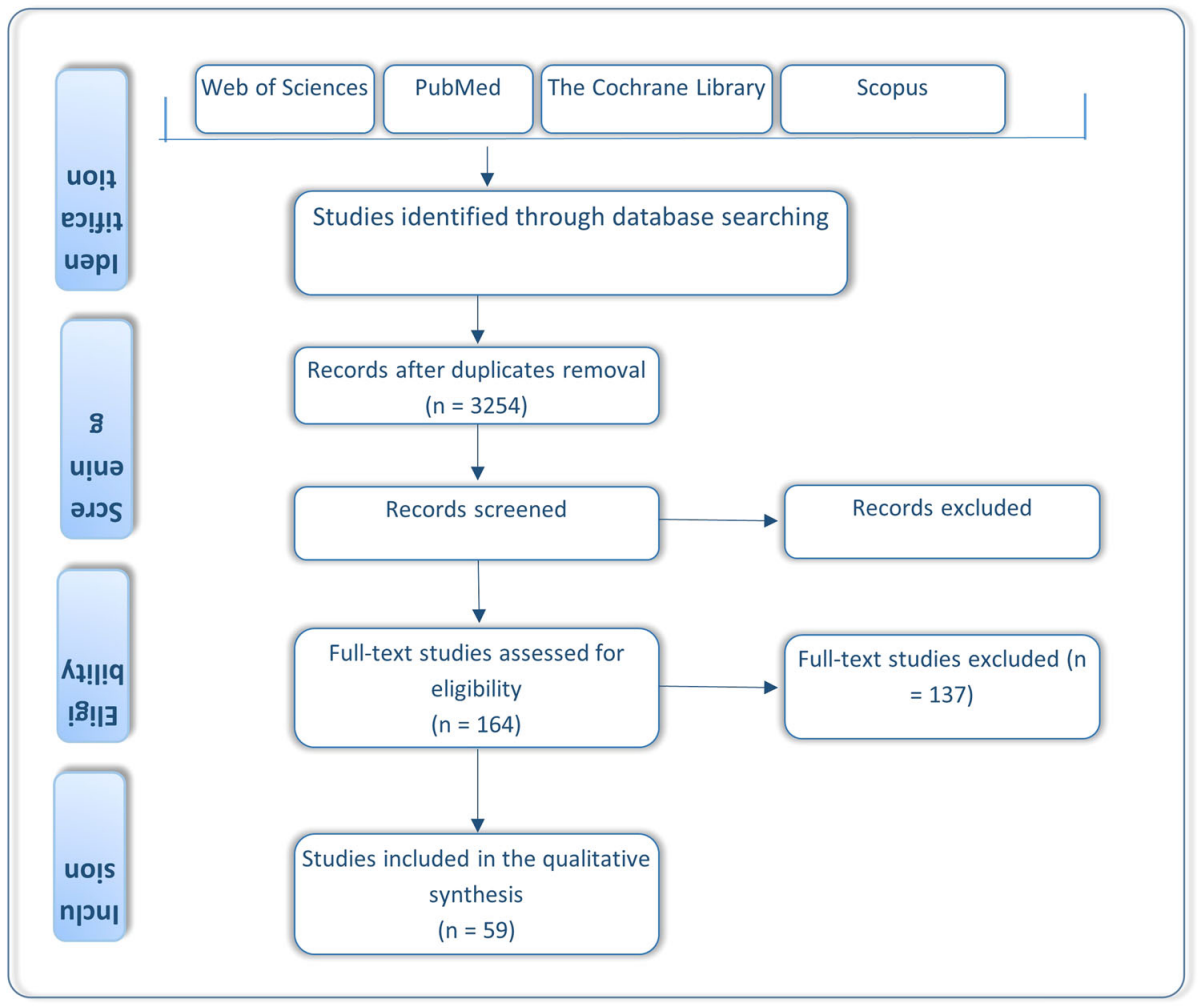
PRISMA flow chart of the study selection procedure.

A systematic electronic search identified a total of 7653 publications, and an additional 1015 articles were identified through backward searching of key papers. Of these, 3254 publications underwent full‐text screening. At this stage, a total of 1002 publications were excluded, and finally, 164 articles met the inclusion criteria. Among these, 59 articles were identified in the pre‐print databases. The results of the search are presented in the PRISMA flow diagram (Figure 2).

### 3.2 Characteristics of Included Studies

**Figure 3:**
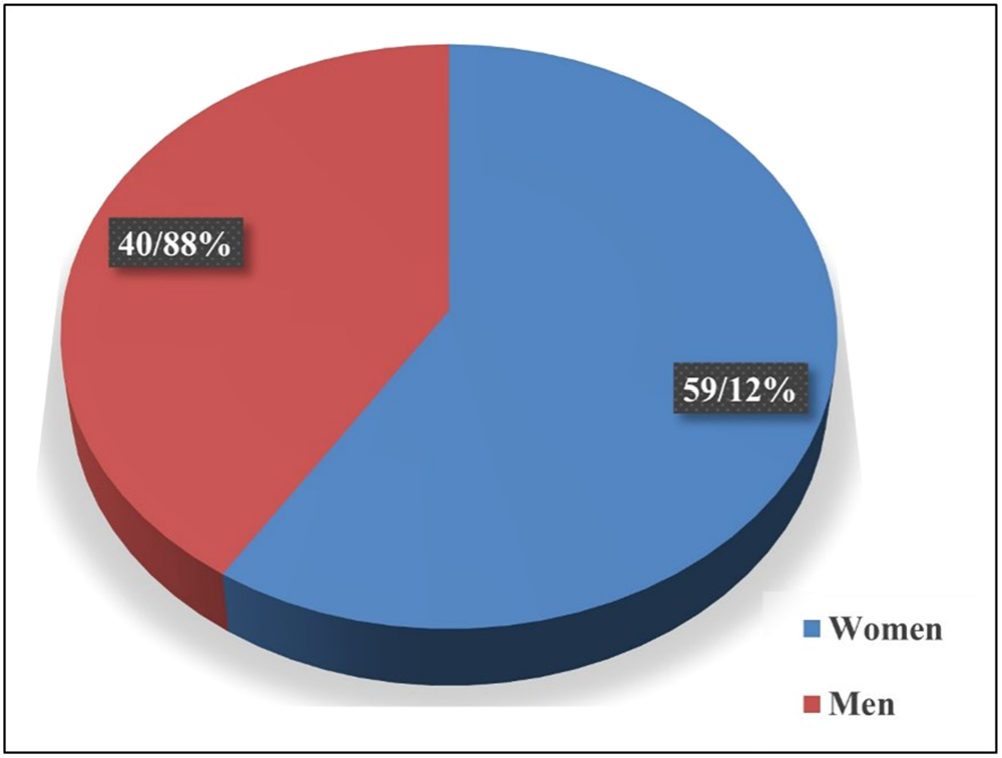
The percentage of men and women.

The studies included in the analysis were conducted in 27 different countries across the continents of Asia (32.2%), Americas (30.6%), Europe (25.8%), Africa (9.7%), the and Oceania (1.6%), with the highest number of studies coming from the United States of America (USA) (*n* = 14), China (*n* = 6), Pakistan (*n* = 5) and Italy (*n* = 4). Of the 59 studies identified, 48 were classified as cross‐sectional, one was designated as qualitative, and one was classified as parallel. The remaining studies did not specify the type of study. A variety of methods were employed to assess attitudes towards the vaccination for COVID‐19 and the associated factors. These included online self‐reported questionnaires and qualitative interviews. Additionally, two studies were conducted in multiple countries. One of these was the study by Timothy D. Dye et al., which involved 173 countries and the USA. Another study by Julio S. Solís Arce et al. included regions such as Africa, South Asia, Latin America, Russia, and the USA [13]. The study with the largest sample size (*n* = 54,727) was conducted in the USA by Salmon et al. [14], while the study with the smallest sample size (*n* = 18) was conducted by Sweety Suman Jha et al. in India [15]. The study sample comprised 58.95% women and 41.05% men, with ages ranging from 16 to 78 years (Figure 3). Among the participants, 69.8% had obtained a university degree, while 31. 2% had not. With regard to the administration of the COVID‐19 vaccine, 36.8% of the participants had received one dose, 10.7% had received two doses, 2.95% had received three doses, and 46.11% had not yet received the vaccine. Furthermore, 3.44% of the studies lacked any information regarding vaccination status. The descriptive statistics of the sample are presented in Table 1 for the reader's convenience. The proportion of participants who accepted the vaccine was 63.3%, while 19.7% exhibited vaccine hesitancy and 17% refused.

**Table 1:** The descriptive statistics of the sample.

No	Outcomes	Education no %	Acceptance %	Hesitancy %	Refuse %	Pregnancy	Dose	Type vaccine	No. of Participants Male % (Mean age ± SD)	Participants Characteristics	Study Design	Survey modality	Country	Reference
1	VH: side‐effects (impact on fertility and pregnancy), Prior COVID diagnosis, vaccine effectiveness VA: trust.	UD: 1745 (74.09) NO UD: 645 (25.27)	84	12	4	NA	1	NA	2491 25 NA	HCWs	CSS	Online	USA	[16]
2	VH: decreasing vaccine confidence, not being afraid of COVID‐19, religious and cultural beliefs, mistrust and less confidence, financial loss	UD: 570 (56.6) NO UD: 1029 (64.3)	88	NA	11	NA	0	NA	1599 49 50 ± 16.23	French‐speakers	CSS	Online	Canada	[17]
3	VH: serious adverse effects, vaccine safety and efficacy, rapid development and implementation of vaccine, rapid development and implementation of vaccine	UD: 415 (100)	63	35	NA	NA	NA	mRNA vaccine	415 NA NA	DS and MS	CSS	Online	USA	[18]
4	VH: short time of building vaccine, fear of vaccine needle, adverse effects, reinfection, type of vaccine, social media, losing family members	UD: 1021 (87.2) NO UD: 150 (12.8)	33	32	35	NA	0	PFZ, AZ, Sinopharm	1171 46 NA	Iraqi Kurdish population	CSS	Online	Iraq	[19]
5	VH: comments from TV, lack of health education about COVID‐19, low income, and age VA: health awareness and education	UD: 384 (80.2) NO UD: 95 (19.8)	40	29	30	NA	0	NA	479 45 NA	Residents of Sukkur City	CSS	Online	Pakistan	[20]
6	VH: serious side effects, lack of trust, and afraid of reinfection	UD: 810 (100)	13	87	0	NA	1,2	PFZ, AZ, Sinopharm	810 40 NA	MS	CSS	Online	Iraq	[21]
7	VH: side effects, unsure of the vaccine, difficulties scheduling vaccination appointments	NA	93	3.52	2.34	NA	1	NA	511 9 42.9 ± 13.6	HCWs	CSS	Online survey	USA	[22]
8	VH: side‐effects, safety, COVID‐19 is not severe enough, confidence in the vaccines, trusting public institutions	UD: 1813 (65.9) NO UD: 938 (31.7)	43	22	35	NA	0	NA	2753 51 NA	Hong Kong residents	CSS	Online	China	[5]
9	NA	UD: 218 (72.6) NO UD: 82 (27.3)	65	28	7	NA	0	PFZ, Moderna, AZ, Janssen	300 24 NA	Spanish citizens	CSS	Online	Spain	[23]
10	VH: safety, efficacy, and side effects, bad experience with a previous vaccination, having enough information	UD: 369 (36.5) NO UD: 642 (63.5)	69	31	0	NA	0	PFZ, Moderna	1011 45 46.9 ± 11.5	Citizens	CSS	Online	Italy	[24]
11	VH: lack of trust in authorities, the vaccine approval process, the vaccines' development velocity, health politics, and the pharmaceutical industry	UD: 4500	92	8	0	NA	1	PFZ, Astra Zeneca	4500 42 NA	HCWs	CSS	Online	Germany	[25]
12	VH: vaccine safety (long‐term side effects).	1450 (100)	64	0	35	NA	2	NA	1450 35 46.3 ± 15.7	CPs	CSS	Online	Italy	[26]
13	VH: safety, side effects, efficacy, and anti‐vaccination beliefs and rumors	UD: 434 (56.7) NO UD: 339 (43.3)	31	69	0	NA	NA	NA	782 56 NA	Bangladeshi	CSS	Face‐to‐face and on‐line	Bangladesh	[9]
14	VH: who had COVID‐19 with severe symptoms, psychological difficulties, Lower levels of trust in media and health information sources and healthcare institutions, agreement with restrictions and higher levels of conspiracy mentality	UD: 487 (24.35) NO UD: 1528 (76.4)	65	28	7	0	1	NA	2,015 49 NA	Italian general population	CSS	Online	Italy	[10]
15	VH: trust CDC, vaccine safety, developed too quickly,	UD: 5252 (53.9) NO UD: 4505 (46.2)	35	0	35	NA	1	Johnson & Johnson	54,727 58 NA	Adults	CSS	Online	USA	[14]
16	VH: safety, fear or dis(trust) in the authorities, or disinterest.	UD: 845 (85) NO UD: 149 (14.9)	36	26	38	NA	1	NA	994 NA NA	PUB	CSS	By phone using CATI	Romania	[27]
17	VH: safety, side effects, effectiveness, effectiveness, religious beliefs, trust and rumor	UD: 65 (9.9) NO UD: 503 (76.7)	84	—	NA	NA	0	NA	655 56 35.13 SD:NA	Rural community	CSS	Face‐to‐face	Bangladesh	[12]
18	VH: fear of adverse reactions	NA	—	.	—	NA	0	NA	18 Male: 8 NA	HCWs and CMs	CSS	IDI	India	[15]
19	VH: trust of government	UD: 2 940 (78) NO UD: 828 (22)	Varicent groups	—	—	NA	0	NA	3768 40 30 ± NA	MEXICAN Population	CSS	Online	Mexico	[28]
20	VH: difficulty in the vaccination request, registration protocols, negative social media reports, bad feelings, rumors, religious beliefs against, poor confidence, allergic reaction concerns, blood clot problems in women	UD: 1525 (100) NO UD: 0 (0)	29	—	71	NA	1,2,3 or more	NA	1525 68 NA	HCW, Academics and Students	CSS	Online	Nigeria	[29]
21	VH: safety and efficacy, lack of information about the disease and vaccine or social media	UD: 507 (90.8) NO UD: 51 (9.1)	80	20	0	NA	1,2	NA	558 54 38.66 ± 9.067	Adults	CSS	Online	Saudi Arabia	[30]
22	VH: side effects and suboptimal efficacy, cost and the need for an annual booster, mistrust of medical personnel	NA	56.2	20	19	NA	1	NA	2,564 90 NA	Jail residents	CSS	Door‐to‐door	USA	[31]
23	VH: vaccine's side effects, long‐term safety and efficacy, Vaccine will not be a solution for COVID	UD: 773 (37.7) NO UD: 1278 (62.3)	92	7	NO	NA	1	NA	2051 49 NA	General Population	CSS	Online and offline	India	[32]
24	VH: confidence and potential personal risk, insufficient evidence, side effects, long‐term effects, speed of the vaccines' development, general safety issues, general effectiveness and lack of knowledge	UD: 476 (100)	94	4	1	NA	1	NA	476 37.5 21 ± 3.4	University student	CSS	Online	UK	[33]
25	VH: side effects, getting coronavirus from the vaccine, efficacy, allergic to vaccines, mentions a conspiracy theory	—	80.3%	—	—	—	1	—	44,260 NA NA	Adults in Lower‐Middle‐Income Countries	CSS	By phone	Africa, South Asia and Latin America, Russia, USA	[13]
26	VH: safety, rushed development, provide protection, reinfection	UD: 238 (81.2) NO UD: 55 (18.8)	63.54	27.42	9	299	1	PFZ, Moderna, Janssen	299 0 NA	Pregnant people	CSS	Live chat service	USA	[34]
27	VH: concern for brand and efficacy, side effects, safety	UD: 248 (100)	88	12	0	7	1	AZ, PFZ, Sino‐Pharm, Sinovac	248 64 NA	HCWs	CSS	In person or online platform	Pakistan	[35]
28	VH: too new, side effects, safety, efficacy, religious objections, not trust vaccines, role of politics in development.	UD: 13112 (75.7) NO UD: 4186 (24.2)	81	19	0	NA	1, 2	PFZ‐BioNTech, Moderna, AZ	21,294 23 NA	Cancer, Autoimmune Diseases	CSS	Online	USA	[36]
29	VH: side effects, safety	UD: 408 (45.0) NO UD:498 (55.0)	69	31	0	NA	1	NA	906 39 NA	PwMDs	CSS	Online or paper‐pencil manner	China	[37]
30	VH: side effects, Vaccine needs more research, expedited vaccine trials, incomplete information, recommendation by the government, lack of information about the vaccine	UD: 3526 NO UD: 247	54	23	13	NA	1	NA	3773 27 NA	Students	CSS	Email	USA	[38]
31	VH: trusted the efficacy and safety. Trust in government and concerns about the impact of vaccine on disease	NO UD: 97 (51.9) UD: 88 (47.1) Not acquired: 2 (1.1)	46	54	0	NA	1	NA	187 NA 64.2 ± 9.2	PD	CSS	Online	China	[39]
32	VH: trust vaccine, illness concern, safety, Trusting vaccine safety	NA	78	0	22	NA	1	NA	168 69 NA	Pennsylvania Autism	CSS	Email	USA	[40]
33	VH: adverse events, efficacy, vaccination fee	UD: 5092 (71) NO UD: 2118 (29)	48	18	34	NA	0	NA	7210 47 NA	General population and HCWs	CSS	Online	Japan	[41]
34	VH: adverse effects, lack of information, afraid of the queue	UD: 185 (60.3) NO UD: 122 (39.8)	94	6	0	NA	1	NA	307 56 24.15 ± 6.8	Population	CSS	Online (social media)	Nepal	[42]
35	VH: side effects, safety, getting COVID‐19 from the vaccine, cost	UD: 3938 (85.1) NO UD: 690 (14.9)	63	37	0	NA	0	NA	4630 33 41.63 ± SD	HCWs	CSS	Google Forms	Iran multicenter	[43]
36	VH: trust, efficacy, safety, painful administration, effectiveness	UD: 205 (50) NO UD: 205 (50)	53	47	0	NA	0	NA	410 43 18‐51+	Population	CSS	Face to face	Pakistan	[44]
37	VH: infected by COVID‐19, efficacy, side effects, trust pharmaceutical companies and public authority, how the vaccine works, using barrier gestures, media and social network	UD: 3089 (100.)	58	25	17	NA	0	NA	3089 30 20.3 ± 1.9	Students	CSS	Online	France	[45]
38	VH: side effects, unsafe, not useful, effectiveness, social media, immune system, religious reasons, unnecessary, Prior exposure, Prior chronic conditions	UD: 343 (26) NO UD:974 (74.0)	60	0	40	NA	1,2	AZ	1325 47 51.1 ± 9.35	VI and non‐VI	CSS	Telephonic interviews	Pakistan	[46]
39	VH: lack of information, people get the vaccine first, needles/injections, not have enough time to take decision, trust the experts ND, risks of the vaccine, efficacy, COVID‐19 is not dangerous	UD: 2,761 (100)	81	14	5	NA	0	PFZ	2,761 28 NA	HCWs	CSS	Email	Canada	[47]
40	VH: side effects, safety and effectiveness, lack of hindsight, short time of production.	UD: 200 (100)	35.5	57.5	6.5	NA	1	PFZ, Moderna	200 46 NA	French‐speaking students	CSS	Interview	France	[48]
41	VH: safety and side effects	UD: 918 (57.4) NO UD: 1094 (42.6)	81	19	0	NA	1	Sinovac, PFZ	2,012 2 39 ± 8.1	Ethnic minorities	CSS	Online	China	[49]
42	VH: risks and safety, confidence, complacency, constraint, calculation, collective responsibility	528 (100)	95	5	0	Exclude	1, 2	PFZ	528 12 NA	HCWs	CSS	Online	Singapore	[50]
43	VH: efficacy, safety	NO UD: 8823 (84.3) UD: 1973 (21.4)	62	9	28	NA	0	NA	10796 42 NA	Mexican population	CSS	NA	Mexico	[51]
44	VH: trust in institutions and public health	UD: 3995 (53) NO UD: 3610 (47)	82	18	0	NA	0	NA	7605 34 NA	HCPs And GDP	CSS	Online (social platforms)	Italy	[52]
45	VH: misinformation and fear, concerns about safety, lack of information, trust issues	UD: 736 (84.5) NO UD: 135 (15.5)	88	0	12	NA	0	NA	871 47 NA	Egyptians	CSS	Online	Egypt	[53]
46	VH: safety and effectiveness, negative information, rushed development, vaccine brand, fear of needles	UD: 217 (100)	56	44	0	0	0	AZ	217 46 NA	MS	CSS	Online (social media)	Sudan	[54]
47	VH: trust in the healthcare system, social media disinformation, Safety,	UD: 4958 (72.0) NO UD: 984 (14.0)	69	0	31	NA	0	NA	6883 46 NA	Social media users	CSS	Online	USA	[55]
48	VH: safety and efficacy	419 (100)	69	21	10	NA	0	NA	419 21 NA	HCWs	CSS	In‐person and online	Poland	[56]
49	VH: baby's health, trust and confidence in vaccine safety	NO UD: 253\253: (100) UD: 0 (0)	59	25	14	287	0	NA	287 0 32.8 ± 5.1	Pregnant women	CSS	Online	Australia	[57]
50	VH: fear, religious reasons, lack of trust, safety, not enough information.	UD: 490 (79.3) NO UD: 128 (20.6)	44	23	33	NA	0	NA	618 49 NA	Adult	CSS	Online	Nigeria	[58]
51	VH: efficacy, adverse events, social, degree of self‐perceived vaccine literacy.	705 (100)	96	4	0	NA	0	Na	705 49 NA	Physicians	CSS	Online	Thailand	[59]
52	VH: medical mistrust, structural barriers, safety and efficacy	UD: 389 (52) NO UD: 353 (48)	60	NA	40	NA	NA	NA	730 51 NA	Americans	CSS	Online	USA	[60]
53	VH: adverse side effects, safety efficacy, short duration of clinical trials, vaccine approve mechanisms.	5312 (100.0)	59	0	41	NA	1	Sinovac, AZ, PFZ, Moderna, Cocktail	5312 16 NA	VHV	CSS	Online	Thailand	[61]
54	VH: Side effects, health, concerns and newness, safety and efficacy, trust	UD: 467 (59.7) NO UD: 315 (40.3)	82	9	5	NA	0	Johnson & Johnson, PFZ, Moderna	789 19 NA	HCWs	CSS	Online	USA	[62]
55	VH: side effects, short period of vaccine development, lack of trust and information, had COVID‐19, after most people take, building immunity through COVID‐19 infection	UD: 434 (82) NO UD: 97 (18)	62	38	0	NA	0	NA	531 60 NA	Adults	CSS	Online	Saudi Arabia	[63]
56	VH: fear of adverse effects, lack of vaccine confidence, Safety	631 (100)	78	11	11	NA	1	NA	631 20 20.08 ± 1.7	Health Care Students	CSS	Online	China	[64]
57	VH: lack of belief in vaccination, efficacy, waiting for a better vaccine, adverse events.	UD: 408 (75) NO UD: 133 (25)	58	42	0	NA	1	Sinopharm, Sinovac, AZ	541 50 NA	General population	CSS	Interview	Pakistan	[65]
58	VH: side effects, trust, rushed development, efficacy, will not stop the infection, consume herbal concoction to Prevent COVID‐19, don't need vaccine	UD: 511 (89) NO UD: 61 (11)	13	0	87	NA	0	NA	572 23 NA	Cameroonians	CSS	In‐person and online	USA	[66]
59	VH: side effects, trust, exaggerated virus impact	UD: 512 (38.79) NO UD: 761 (61.26)	71	15	14	NA	0		1284 57 NA	New Zealanders	CSS	Online	New Zealand	[67]

Abbreviations: AZ, AstraZeneca; CMs, community members; CPs, community pharmacists; CSS, cross‐sectional stud; DS, dental students; GDP, general adult population; HCPs, healthcare professional; HCW, healthcare workers; IDI, in‐depth Interview; MS, medical student; MS, medical students; No UD, no university degree; Non‐VI, non‐vaccinated individuals; PD, Parkinson's Disease; PFZ, pfizer; PUB, public; PWMDs, persons with mental disorders; UD, university degree; VA, vaccine acceptance; VH, vaccine hesitancy; VHVs, village health volunteer; VI, vaccinated individuals.

### 3.3 Rates of COVID‐19 Vaccine Hesitancy and Acceptance

**Figure 4:**
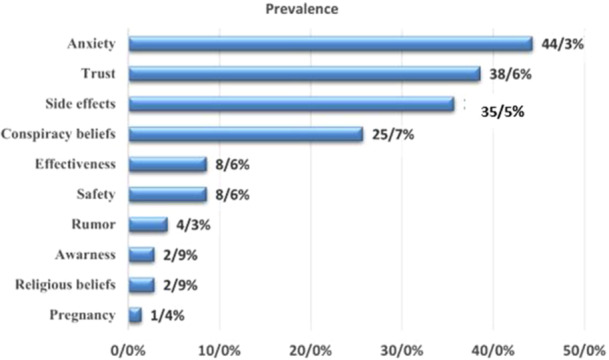
Factors influencing COVID‐19 vaccine acceptance or hesitancy.

The results of the acceptance rates for the novel COVID‐19 vaccine, stratified by country, are presented in Table 1. Among the general public, the highest rates of acceptance of the vaccine were observed in Thailand (96%), while the lowest rates were recorded in the USA and Iraq (13%). The proportion of participants exhibiting vaccine hesitancy in the reviewed studies ranged from 0% to 87%. 35.5% (21/59) of the included studies feared vaccination due to the occurrence of adverse effects, including long‐term, short‐term, and unpredictable effects. However, 13% of participants experienced serious vaccination side effects and 33.9% experienced minor adverse effects. Additionally, a total of 59 included studies reported concerns related to safety (8.6%), efficacy (8.6%), and 14.2% for other reasons (such as religious beliefs, pregnancy, etc.). Approximately 10.2% of the studies identified concerns regarding the accelerated development of the vaccine and the limited time spent in clinical trials. The most commonly reported common adverse effects associated with the administration of the Covid‐19 vaccine include fever, fatigue, headache, nausea, dizziness, muscle pain, skin rash and swelling, restlessness, injection site pain, joint pain, purpura, erythema, pyrexia, dyspnea, and chills. Serious side effects include blood clots, thrombocytopenia, anaphylaxis shock, seizures, infertility, and cardiac infarction may occur, these are rare. Amongst patients who reported unfavorable cardiovascular events, the majority were male. Furthermore, a greater proportion of males than females reported adverse effects, including chest discomfort, dyspnea, and palpitations. These adverse effects were observed with greater frequency following the administration of the second dose of the vaccine compared to the first. Figure 4 presents a list of factors that influenced the acceptance or hesitancy regarding the use of the COVID‐19 vaccine, as observed in the study. It is important to note that the lack of standardization precludes the possibility of comparing data over time and space. The most salient factors related to vaccine hesitancy are structural and include the following:

Social movements of public health vaccine opposition have become increasingly bigger and contributed to the increase in the percentage of the population, predominantly in the EU and USA, who have refused vaccination efforts in recent years. Barriers to access ability, including vaccine delivery time, location, and cost related to socioeconomic inequalities and marginalization. Safety concerns, including concerns about vaccine ingredients and belief that the vaccines are directly responsible for some deaths. The findings of this study revealed several motivating factors that encouraged participants to accept the vaccination against the novel coronavirus, COVID‐19. These factors included an enhanced perception of the risks associated with COVID‐19 infection and trust in the healthcare system. The level of health awareness and the quality of the educational resources available were identified as significant motivators for vaccine acceptance. Interactions with individuals who had contracted the disease reinforced participants' perception of the severity and high infectiousness of the disease. A substantial body of evidence attests to the efficacy of vaccination in mitigating the risk of severe illness, hospitalization, and mortality from COVID‐19 infection. The decision to be vaccinated was influenced by several factors, including concern for the well‐being of family and loved ones, personal risk perception of the virus, and endorsement or approval by authoritative bodies such as the Food and Drug Administration (FDA). Furthermore, reasons pertaining to work also influenced the decision to accept the vaccine.

## 4 Discussion

In the latter part of 2020 and the early months of 2021, vaccines against the novel coronavirus received approval for general use in countries across the globe [68]. The WHO has identified vaccine hesitancy as one of the ten most significant global threats to public health [69]. One of the most crucial and efficacious methods for addressing vaccine hesitancy is to identify the underlying reasons for this phenomenon. It is of the utmost importance to gain an understanding of the reasons behind and the means of effectively addressing vaccine hesitancy. In light of the global dissemination of COVID‐19 and the urgent necessity for efficacious vaccination to avert the associated illness, it is disconcerting that numerous individuals evince reticence to receive the COVID‐19 vaccine due to misgivings. The objective of this study was to investigate the prevalence of vaccine hesitancy and acceptance on a global scale, as well as the underlying reasons. The findings of this study corroborate the assertion that trust in the healthcare system and an elevated perception of risk associated with COVID‐19 infection are pivotal factors in fostering vaccine acceptance. Those with a history of unfavorable vaccine outcomes exhibited elevated levels of hesitancy. In accordance with the Strategic Advisory Group of Experts (SAGE), the determinants of vaccine hesitancy can be classified into three principal categories: contextual effects, individual/group effects, and vaccine‐specific issues. Socio‐demographic factors associated with vaccine rejection include being a parent, being a homemaker, being retired, being unemployed, having a child with an underlying disease, being under the age of 60, belonging to the Black race, having lower education levels, residing in a rural area, having low income, and lacking health insurance [70]. It is important to note, however, that some studies excluded participants who selected the “unsure” option instead of outright rejecting the vaccine, resulting in lower reported rates of vaccine hesitancy [71]. Categorizing individuals into only “anti‐vax” and “pro‐vax” is not accurate, as many people fall within a spectrum between these two categories [72]. Previous vaccination experiences also impact individuals' willingness to receive future vaccines. Those who have received the seasonal flu vaccine in the past are more likely to accept the COVID‐19 vaccine [73, 74]. In another systematic review conducted during the first year of the coronavirus pandemic, vaccine acceptance was investigated. The findings demonstrated that older age, being male, higher education, a history of influenza vaccination, perceiving a higher risk of COVID‐19 compared to vaccination, and having high trust in government‐provided information were associated with increased vaccine acceptance [75]. One reason for vaccine nonacceptance is the skepticism some individuals have regarding the existence of the coronavirus [76]. Others perceive the virus to be similar to the influenza virus, considering it nonlife‐threatening and not necessitating vaccination [69]. Continued acceptance of the COVID‐19 vaccine is crucial due to the waning immunity against COVID‐19 and the constant emergence of new viral strains (lineages) that may potentially evade the effectiveness of current vaccines [77, 78, 79, 80]. During the COVID‐19 pandemic, the world became increasingly physically distant, with lockdown measures such as social distancing, disbanding of public gatherings, and remote work environments instituted in many countries worldwide. Consequently, social media grew to fulfill a critical role as a source of social news and the primary information outlet for governments and health organizations [27]. Different social media facilities make it easy for individuals to find health information. However, antivaccine groups were active on social media and spread misinformation, which also influenced willingness to vaccinate [12] Moreover, social media offers direct communication between HCPs and patients, is known to reduce vaccine concerns and improve overall uptake and has made health able to produce news content and public service announcements. This content should be designed to effectively educate and reassure broad, diverse audiences regarding key vaccine‐related topics [31, 32]. Furthermore, the media plays a role in fostering vaccine hesitancy through the dissemination of content, including programs, discussions, and headlines that are either contentious or open to interpretation [81]. The circulation of reliable and unreliable information regarding the COVID‐19 vaccination on social media platforms can have a significant impact on the acceptance and hesitancy of the vaccine. The dissemination of false information on social media platforms such as Facebook and Twitter can have a significant impact on individuals' decisions regarding the acceptance or rejection of the vaccine [82]. One particularly pervasive issue that has been extensively disseminated in virtual spaces and social networks is the conspiracy theory positing a causal link between infertility and the COVID‐19 vaccine. This theory has served to further exacerbate existing doubts about the safety of the vaccine [83]. Several articles have indicated that women exhibit higher levels of vaccine hesitancy compared to men [68, 70, 84]. There can be various reasons contributing to the higher willingness of men to be vaccinated. Firstly, sampling bias in studies may play a role [85], Secondly, women may experience less social support and display a lower willingness to accept healthcare recommendations and preventive measures [75]. Recent data from the national vaccination registry in Austria show a narrowing of the gender gap in vaccination. But with a closer look, it is clear that there are gender differences in vaccine absorption in some subgroups of young people and the unemployed. In 25‐year‐old unemployed men, the rate of vaccine uptake is 5% higher than that of their female counterparts. Maybe the reason for this is women's concerns due to their reproductive age or the responsibility of taking care of the child [86]. A systematic review of the factors influencing vaccine acceptance in the USA identified male gender and university education or higher as the most significant predictors of acceptance. The lowest rates of acceptance were observed among Black non‐Hispanic individuals, pregnant and breastfeeding women. Conversely, the most positive attitudes towards the vaccine were seen among White individuals, Asians, and those over 45 years old. Factors contributing to hesitancy included uncertainty about the vaccine's effectiveness and side effects, religious reasons, and a lack of trust in the healthcare system [85]. Almost all the studies included in this research, as well as various review studies, concur that younger individuals exhibit higher levels of vaccine hesitancy compared to older individuals [68, 70, 75, 85, 87]. Several reasons can be considered for this trend. Firstly, younger people generally experience milder cases of COVID‐19, which may lead them to perceive a lower personal risk associated with the disease. Secondly, younger individuals often possess a higher tolerance for risk, which can result in a diminished understanding of the potential dangers associated with not receiving the vaccine and consequently contribute to the spread of the disease within society [88, 89].

The racial background of individuals has been identified as a factor associated with vaccine hesitancy in numerous studies. According to these studies, black or nonwhite individuals tend to exhibit higher levels of vaccine hesitancy compared to others [70, 71, 85, 87, 90]. Several factors can be considered to explain this phenomenon, including racism, discrimination, and mistreatment within healthcare systems, which significantly impact these individuals [91]. In the systematic review conducted by Troiano and colleagues, the factors influencing vaccine hesitancy were investigated. It was found that African Americans, similar to their historical patterns regarding the flu vaccine, exhibited high levels of hesitancy. Additionally, unemployed individuals, those with low income, and individuals with religious affiliations showed lower acceptance rates [87]. Religious reasons underpinning the vaccine hesitancy were identified for many religious groups, including Protestants, Catholics, Jewish, Muslims, Christians, Amish, Hinduist and Sikhist. For instance, porcine or non‐halal ingredients content of vaccines was the main barrier identified in Muslim populations. A study carried out in Guinea revealed that 46% of Muslims and 80% of religious leaders considered that vaccination was not allowed during the Ramadan [92]. Within the Muslim community, the belief that the vaccine is considered haram (forbidden) is a significant reason for vaccine rejection [93]. This concept of haram gained momentum in 2011 with the spread of anti‐vaccine propaganda, leading to the belief that vaccines were part of a conspiracy by Western countries to sterilize girls [81]. Furthermore, the presence of the belief that “God did not take any medicine” has contributed to vaccine hesitancy among individuals who hold strong religious beliefs. Additionally, some people associate vaccines with Satanism, further intensifying their hesitancy [94]. A study conducted in Nigeria revealed that the primary reason for hesitation is the fear of bioterrorism. Indeed, the Nigerian populace is of the opinion that the vaccine will be employed as a biological weapon against them, with the objective of reducing the population size through the insertion of microchips into the body [95]. The rapid development of the vaccine and the limited time spent on clinical trials accounted for 10% of the studies. Additionally, individuals with a history of adverse outcomes from previous vaccinations tended to exhibit higher levels of vaccine hesitancy. Skepticism about vaccine efficacy, based on stories about new variants, breakthrough infections, and people who were already vaccinated against COVID‐19 passing the virus on to others; risk versus benefit, with participants suggesting that COVID‐19 vaccines are unnecessary, are riskier than the virus itself, or are only for the people most vulnerable to infection. A paucity of trust in the integrity and competence of institutions such as physicians, public health authorities, and the government; and concerns about the potential for disparate health outcomes based on race or ethnicity have been reported.

In a study conducted in 2021 regarding parents' vaccine hesitancy, it was found that parents also had reasons for vaccine hesitancy for their children which stemmed from inadequate and insufficient information provided by doctors, as well as concerns about the safety of the vaccine [96]. In a systematic study conducted in 2021 on vaccine hesitancy among healthcare workers (HCWs) worldwide, the prevalence of vaccine hesitancy ranged from 4.3% to 72%, with an average of 22.51% [17]. The most significant reasons for vaccine hesitancy among HCWs were concerns regarding safety, effectiveness, and side effects. The acceptance of the vaccine was higher among men, older individuals, and doctors [68]. In a study conducted in the United Kingdom, vaccine hesitancy was higher in women (21.0% vs. 14.7%), younger age groups (26.5% in 16–24‐year‐olds vs. 4.5% in those aged 75+), and those with lower educational attainment (18.6% no qualifications vs. 13.2% graduates). Vaccine hesitancy was high among black (71.8%) and Pakistani/Bangladeshi (42.3%) ethnic groups [7]. In another study conducted in the United Kingdom, reasons for vaccine rejection, in addition to the aforementioned factors, were attributed to a lack of transparency in vaccine production and a lack of trust in vaccination staff [97]. In a study conducted by Biswas et al., the vaccine hesitancy rate was reported to range from 10% to 57.8% [68]. In Joshi et al.'s study, which covered data until mid‐December 2020, it was revealed that several factors influenced vaccine acceptance, including age, sex, race, education, employment, income level, marital status, parenthood, and geographic location. The study demonstrated that the highest vaccine acceptance rates were observed in Indonesia (93%), China (91%), and England (89%), while the lowest acceptance rate was recorded in the United Arab Emirates (UAE) at 22% [70]. In a systematic review that encompassed articles until December 25, 2020, the highest vaccine acceptance rates were found in Ecuador (97%), Malaysia (94.3%), Indonesia (93.3%), and China (91.3%), whereas Kuwait (23.6%) and Jordan (28.4%) reported the lowest acceptance rates [1]. Additionally, Hong Kong was also found to have low confidence in vaccines. Malaysia was reported to have the highest vaccine acceptance rate [98]. In a global survey encompassing 19 countries, it was found that Brazil had the highest vaccine acceptance rate at 85.3%, while Russia had the lowest acceptance rate at 54.8% [99]. According to Sallam and colleagues' study, the highest vaccine acceptance rates were observed in Nepal and Vietnam (97%), Nigeria (93%), Ethiopia and Tanzania (92%), and Canada (91%). On the other hand, Iraq had the lowest acceptance rate at 13% [100]. The results of various systematic studies conducted in different time frames and by different researchers on the level of vaccine hesitancy around the world have revealed significant regional and national variations. These variations are evident across different geographical regions, economic status, political and cultural status. However, the specific factors that have led to these doubts and the relationship between these factors and the occurrence of doubt remain unclear. Consequently, it is recommended that a more comprehensive understanding of the situation in each country with a high rate of vaccine skepticism be sought, taking into account the economic, political, social, cultural, and other relevant factors.

**Figure 5:**
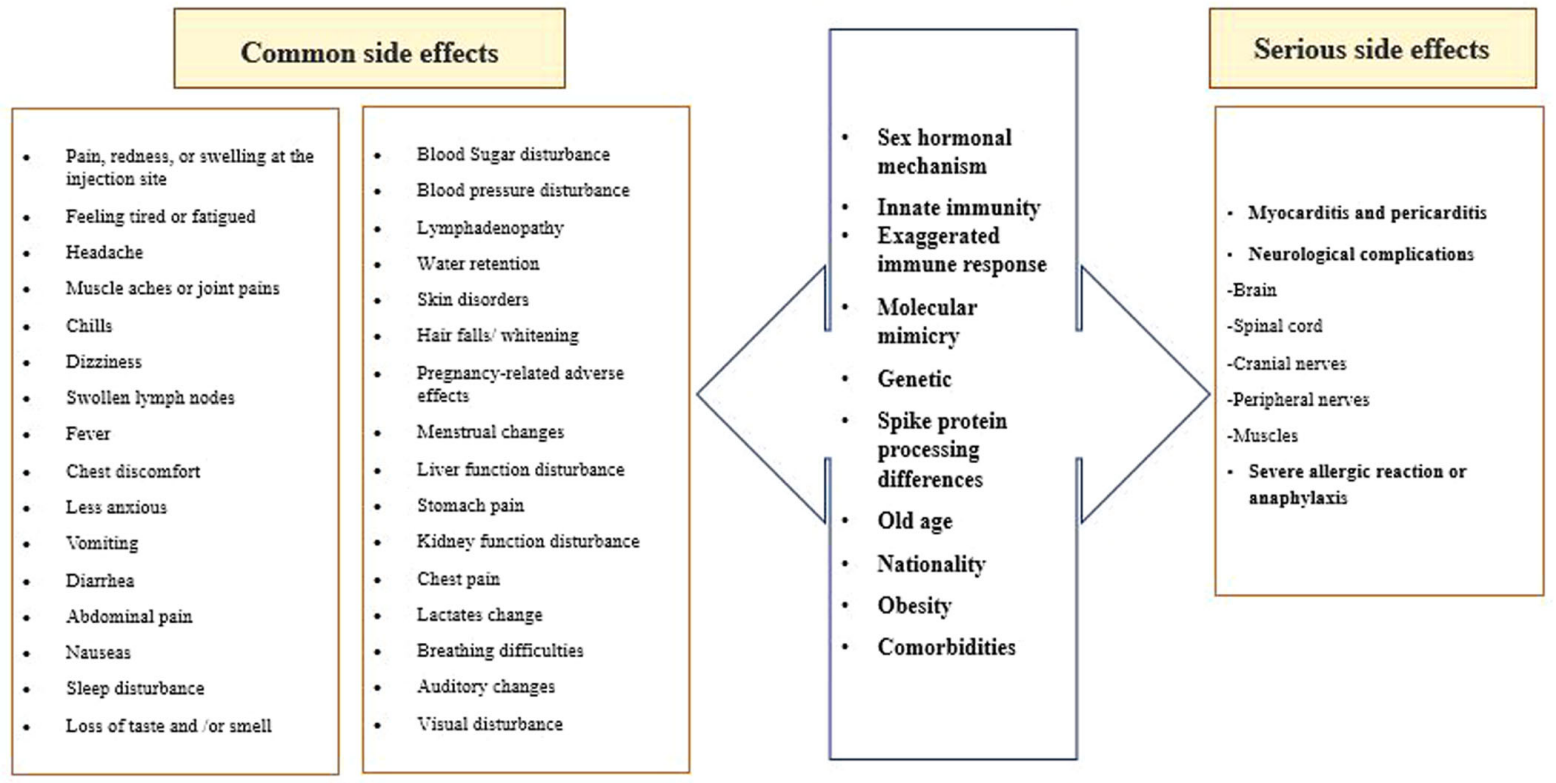
COVID‐19 vaccine‐related adverse events and factors affecting them.

In the present study, the proportion of individuals exhibiting vaccine hesitancy ranged from 0% to 87% across the various studies. The findings of this study indicate fear of potential side effects is one of the main reasons for vaccine hesitancy. The immune response to the COVID‐19 vaccine depends on factors such as vaccine type, age, gender, body mass index (BMI), pre‐vaccination comorbidities, spike protein processing differences, nationality, sex hormone, and previous COVID‐19 infection associated with side effects. The list of side effects and factors affecting them are shown in Figure 5. Among these potential adverse effects, it is possible to cite instances of blood clotting and an impact on future fertility [35, 36]. It has been demonstrated that acute COVID‐19 infection affects sperm parameters; however, the effect of the vaccine on this remains to be determined. Consequently, a significant proportion of the population has concerns regarding the potential for infertility and the adverse effects of future pregnancies [101, 102]. Several reports of thrombocytopenia with thrombosis, most notably cerebral venous sinus thrombosis or cerebral venous thrombosis (CVT) within 28 days of vaccination, have been associated with covid 19 vaccines. Some risks are associated with COVID‐19 vaccinations, but no vaccination is entirely safe. Generally, short‐term adverse effects of the COVID‐19 vaccines present with mild symptoms. The most common symptoms are localized pain and swelling at the injection site, fever, headache, myalgia, and chills. Cases of thrombosis, notably CVT, are mostly seen with the adenoviral vector vaccines. Adverse effects such as myocarditis, glomerular diseases, and cutaneous eruptions are seen with the mRNA vaccines (Pfizer‐BioNTech and Moderna). Myocarditis has been recognized in young adults with males affected more often than females. The important potential pathogenesis of mRNA COVID‐19 vaccine‐induced myocarditis is molecular mimicry between the spike protein of Covid‐19 and self‐antigens, gender, and genetics (genes encoding HLA factors). The higher incidence of COVID‐19 vaccine myocarditis in young males may be explained by significantly different sex hormone‐related immunological response between genders. For example, estrogen in women reduces levels of cardiac inflammation during myocarditis by activating a Th2‐type immune cells response, stimulation of Foxp3+ regulatory T cells, regulatory M2 macrophages, and inhibition of pro‐inflammatory T cells. In contrast, testosterone in males produces a robust pro‐inflammatory Th1 response during myocarditis by mast cell activation, inhibition of anti‐inflammatory cell populations, and increased complement pathways. Studies have shown myocarditis is associated increase of pro‐inflammatory cytokines IL‐1, and IL‐18. In this study was shown that the severity of side effects after the second dose is higher than the first dose of vaccination. This can be due to the intensity of the immune system response in the second exposure to the vaccine or might be associated with perceived severity among study participants. In the present study, the highest acceptance rate was reported in Thailand at 96%, while the lowest acceptance rates were observed in the USA and Iraq (13%). It is important to note that vaccination rates are not necessarily correlated with vaccine hesitancy. For instance, the UAE reported a high vaccination rate among its population (39.3%) but also showed a high rate of vaccine hesitancy [71]. Conversely, Canada had low vaccine hesitancy, but its vaccinated population was only 3.3% as of May 2021, indicating a very low percentage [103]. The most effective components in reducing vaccine hesitancy include: (1) targeting specific groups, (2) increasing knowledge about the vaccine, (3) improving access and convenience of vaccination, (4) considering mandatory vaccination, (5) involving religious and political leaders to encourage vaccine acceptance, and (6) integrating new vaccine evidence and knowledge into routine practice [72, 100]. To address vaccine hesitancy, it is essential that health experts, public institutions, representatives of the labor force, unions, professional associations and policymakers engage in collaborative efforts [72]. The implementation of public health campaigns by organizations such as the Centers for Disease Control and Prevention (CDC) or the WHO can assist in the alleviation of the mistrust that is often held by the general public regarding health organizations and medical professionals. The endorsement of the vaccine by political and religious leaders can significantly contribute to positive vaccine acceptance, as their substantial influence may facilitate this [104]. Another effective method is the implementation of mandatory vaccination policies in workplaces [105]. Furthermore, addressing economic disadvantage by reducing the cost of vaccination or even making it free can help to overcome one of the reasons for vaccine hesitancy. Furthermore, conducting comprehensive research on vaccines and mitigating their adverse effects can enhance public acceptance of vaccines [104]. A vaccination program can be expected to achieve its planned goals when it is supported by evidence of the safety of the vaccine in question, high levels of public acceptance, and demographic coverage that is appropriate to the context in which the vaccine is being introduced. To address vaccine hesitancy, it is essential that evidence‐based strategies are implemented at the organizational, interpersonal, and individual levels within clinical organizations [106]. According to a study conducted by Sallam and colleagues, vaccine hesitancy rates vary across different continents. The African continent exhibits a low rate of vaccine acceptance, while the Asia and Pacific region generally has a high rate of vaccine adoption. Central Asian and Eastern European countries experience high vaccine hesitancy due to a knowledge gap. Latin America and the Caribbean show an acceptance rate of over 70%. The Middle East and North Africa region has low levels of vaccine acceptance, although countries like Israel and the United Arab Emirates (UAE) have achieved high acceptance rates. Western and Central Europe also report a high rate of vaccine hesitancy [100]. Shakeel et al. reported the average vaccine acceptance rates in different continents as follows: South America 78.44%, Australia 77.96%, North America 68.32%, Europe 66.54%, Asia 63.01%, and Africa 56.59% [83]. The African continent, which is among the lowest‐income regions of the world, exhibits a general reluctance to accept vaccines for a number of reasons. One of the reasons for this is the historical misuse of medical research and colonial vaccines, which has resulted in a loss of trust in current vaccines. Furthermore, the absence of precise and culturally appropriate comprehension, coupled with the dissemination of misinformation about vaccines, has intensified the skepticism surrounding vaccination on this continent [107]. The acceptance rate of vaccines is influenced by changing circumstances over time. In Joshi et al.'s study, it was demonstrated that the global vaccine acceptance rate was 86% until March 2020, decreased to 54% by July 2020, and then increased to 72% by September 2020. Vaccine hesitancy has reduced over time in some countries, such as the United States [70].

The role of the type of vaccine (attenuated, killed, and recombinant) in causing vaccine hesitancy is not yet clear. Therefore, more studies are needed to inform policy decisions [108]. Global myths and false beliefs about the COVID‐19 vaccine are related to lack of awareness and conflicting beliefs about its effectiveness, side effects, and purpose [109, 110, 111, 112]. The most crucial factors are the establishment of trust in the healthcare system and the enhancement of awareness and education, with the objective of promoting vaccine uptake. It is imperative that the underlying factors contributing to vaccine hesitancy be thoroughly examined, including geopolitical, religious and cultural, and demographic elements [100]. Clear and honest communication is effective in building public trust and promoting positive health behaviors, such as vaccination [113]. Assessing the impact of vaccine hesitancy on vaccine uptake requires time and careful consideration [71]. Several factors influence the uptake of vaccines. Firstly, there are logistical and administrative challenges associated with establishing vaccine distribution systems. Secondly, issues related to vaccine production capacity can impact availability. Thirdly, the cost‐effectiveness of the vaccine plays a role. Lastly, the global allocation of vaccines becomes crucial, particularly in the context of limited supplies [114]. When planning future COVID vaccination programs, two key aspects should be given special attention. First, if the COVID vaccination can effectively prevent virus transmission, it is recommended to vaccinate at least 60‐70% of the population [115]. Second, if the scenario “COVID vaccine only reduces the severity of the disease” holds true, identifying target groups for vaccination becomes essential [71]. Therefore, it is important to study vaccine hesitancy within subgroups with high mortality rates [71].

Since the emergence of the COVID‐19 pandemic and the efforts to produce an effective vaccine against it, many articles have investigated the reasons for rejecting and accepting the vaccine. The abundance of available articles has prompted some researchers to collect the available data and present a general result. Therefore, many articles have been published in the form of a systematic review (Table 2). In this article, we selected some articles by providing keywords and limitations and performed the necessary analysis. Through the investigations, it was found that the present study and the previous systematic reviews differ from each other from several points of view, and this has also caused the difference in the results. For example, the studies were published in different years, the number of included articles, and the inclusion and exclusion criteria are different. There were also differences in the type of included studies. In some, only one type of study is included, but in others, the type of study is not important. Despite the many differences, the reasons for rejecting and accepting vaccines are very similar, with concerns about the safety, efficacy of vaccines, and the existence of side effects being at the top of the list.

**Table 2:** Overview of published systematic review studies.

Author	Year of publish	Country	Number of incoming articles	Lowest COVID‐19 vaccine acceptance rates (%)	Highest COVID‐19 vaccine acceptance rates (%)	Searched databases	Inclusion criteria	Exclusion criteria	Reasons for rejecting or hesitating the vaccine	Reference
Sangeeta Dey	2023	India	46	40	92	PubMed, Scopus, and GS	Original studies on any aspect of the COVID‐19 vaccine, Pub in Eng, based on data from India	Studies on vaccine acceptance/hesitancy, healthcare workers' perspectives, CTs, REVs, commentaries, editorials, letters, case reports, studies on COVID‐19 impact, postvaccination severity, and those conducted outside India	SE, SFTY, clinical‐term efficacy, distrust in manufacturers, health systems, RD, lack of CTs, lack of awareness, personal health factors, cultural/personal beliefs, anti‐vaccine attitudes, fear of vaccines, effectiveness concerns, illness experiences	[116]
Jember Azanaw	2023	Sub‐Saharan Africa.	29	29.08	76.81	GS	Cross‐sectional studies, Pub in Eng, investigating COVID‐19 vaccines, using standardized and validated tools	Nonrelevant articles, incomplete reports, low‐quality studies or studies with a score of 0–4 points	Misunderstandings about vaccine SFTY and efficacy, low confidence in government and HA, local cultural and traditional beliefs	[117]
Mohd Noor Norhayati	2022	Malaysia	172	52	74	PubMed, Embase, Medline, WoS, GS, and Cochrane Library databases	Only original research articles, published between 2020 and July 2021, with full text available	Non‐English articles, case reports, conference papers, abstracts, REVs, and qualitative studies	Perceived SE, distrust in healthcare system and policies, RD, media influence and misinformation, confusion from repeated exposure to pandemic and vaccine information	[118]
Dechasa Adare Mengistu,	2022	Ethiopia	68	15.4	95.6	MEDLINE (PubMed)	COVID‐19 vaccine acceptance studies, quantitative studies, Pub in Eng	Non‐free articles, high‐risk bias studies, articles without relevant outcomes, noninclusion criteria studies, and includes peer‐reviewed articles published from March 2020 to June 2022	Fear of SE, distrust in government and healthcare systems, religious or cultural beliefs, lack of access to accurate information, misinformation and conspiracy theories, concerns over RD, preference for natural immunity	[119]
Wafa Abu El Kheir‐Mataria	2023	Egypt	13	4.9	91	PubMed, Scopus, WoS, Embase, CINAHL, and GS	Studies (Dec 2021 ‐ Feb 2022) on COVID‐19 acceptance or hesitancy in low‐ and middle‐income countries, using quantitative or mixed methods, focusing on parents and caregivers with vaccine access, Pub in English	Targeting non‐parents, non‐English languages, high‐income countries, or using qualitative methods	Concerns about vaccine efficacy, SFTY, and potential SE	[120]
Fidelia Cascinia	2021	Italy	209	21.4	91	PubMed, WoS, and Cochrane	Conducted in English, investigating attitudes or barriers to COVID‐19 vaccine acceptance, using validated techniques, peer‐reviewed, with full text available. Includes studies on the general population or healthcare workers specifying target population, location, and sample size	Non‐peer‐reviewed studies, non‐English publications, studies without publicly available full text, and duplicate reports	Fear of SFTY and SE, RD, LoT in public HA, and desire for normalcy.	[108]
Sultan Mahmud	2021	Serbia	78	15.40	58	Library	Conducted in English, investigating attitudes or barriers to COVID‐19 vaccine acceptance, using validated techniques, peer‐reviewed, with full text available. Includes studies on the general population or healthcare workers, specifying target population, location, and sample size	Non‐observational studies, those missing vaccine acceptance rates, those without specified location/country, and those without available full‐text	Misinformation, LoT, concerns about SFTY, cultural beliefs, accessibility issue	[121]
Esteban A. Alarc´on‐Braga	2022	USA	18	68.8	88.4	PubMed and WoS	In Latin America and the Caribbean, assess COVID‐19 vaccine acceptance in adults, focusing on demographics, beliefs, and trust	Case reports, scoping REVs, narrative REVs, systematic REVs, and conference abstracts	Fear of SE, distrust in local health systems, misinformation or fake news on social media, political issues, local concerns	[122]
Malik Sallam	2021	Bangladesh	30	23	97	PubMed, MEDLINE, WoS, and GS	Peer‐reviewed PubMed articles, surveys of the general population, health workers, students, or parents, focusing on vaccine acceptance/hesitancy, Pub in Eng	Preprints; articles not on vaccine acceptance/hesitancy; non‐English publications.	LoT in vaccine SFTY and efficacy, insufficient clear and timely communication from trusted sources	[1]
Muhammad Mainuddin Patwary	2022	Peru	33	42.6	76.7	PubMed, Scopus and WoS	Surveys with no population restrictions, descriptive/observational studies, questions on COVID‐19 vaccine acceptance/hesitance, conducted in low‐middle‐income countries, peer‐reviewed, Pub in Eng from January 2020 to August 2021	Non‐focused articles on vaccine acceptance/hesitancy Literature/systematic REVs, meta‐analyses Unpublished data, books, conference papers Editorials, commentaries, non‐full‐text case reports, studies	SFTY and efficacy, RD, reliability and religious inhibition, risks and benefits of vaccines	[123]

Abbreviations: CTs, clinical trials; GS, Google Scholar; HA, health authority; LoT, lack of trust; Pub in Eng, published in English; RD, rapid development; REVs, reviews; SE, side effect; SFTY, safety; WoS, web of science.

## 5 Strengths and Limitation

Like other systematic studies, this study also has its limitations. One limitation worth mentioning is the variation in demographic characteristics across different regions, which can impact the percentages of vaccine acceptance and hesitancy. Additionally, there may be biases present in certain studies, which can influence the overall quality of the research. Furthermore, the studies included in this analysis were conducted at different time periods, and in many cases, the impact of time is not accounted for. Given that vaccine development is a rapidly evolving field, the results can be influenced by the timing of publication. It is worth noting that this study exclusively included full‐text studies, and pre‐print studies were not considered. Furthermore, only studies published in English were included, resulting in the potential omission of relevant studies conducted in other languages. Most of the studies included in this systematic review were cross‐sectional in nature, which solely gather information at a specific point in time and do not establish causation [124, 125, 126]. A common issue in surveys is that participants often self‐select to take part, which may introduce systematic differences compared to the general population [127]. Lastly, the majority of the included studies relied on self‐reported surveys, which are subject to social desirability and recall biases [128, 129, 130].

## 6 Summary and Prospect

The COVID‐19 virus represents one of the most significant challenges currently facing the global community. The development of an effective vaccine may prove to be the most effective solution to address this global problem. However, despite the existence of widespread hesitations surrounding the use of the vaccine, it is clear that the path to resolving this issue will not be without obstacles. The acceptance of the vaccine is influenced by a number of factors, including age, gender, and various socioeconomic variables [6, 131]. It is of the utmost importance to eliminate vaccine hesitancy or increase vaccine acceptance. This should be pursued through various approaches in collaboration with regulatory and healthcare organizations. Given the complex and multifaceted nature of vaccine hesitancy in the context of the ongoing pandemic, it is crucial to assess the existing literature on this phenomenon and its underlying determinants. The present study offers a comprehensive examination of the challenges hindering the nationwide vaccination campaign against COVID‐19, providing valuable insights and recommendations for addressing these obstacles. It is particularly noteworthy that such studies will prove invaluable in future epidemics. Additionally, systematic reviews of studies in this field can help policymakers and stakeholders gain awareness of the determinants that can enhance community‐based interventions related to vaccination. To carry out a nationwide and efficient vaccination against COVID‐19 or any such epidemic disease, it is necessary for all related organizations to remove the existing obstacles so that people's confidence increases and the desire to use the vaccine increases. Although the emergency phase of the COVID‐19 pandemic may be ending, the possibility of future outbreaks of communicable diseases remains. Therefore, knowledge about similar subjects is a valuable approach for health experts and decision‐makers.

## Author Contributions

**Atieh Darbandi:** conceptualization. **Parisa kiani:** investigation. **Roya Ghanavati:** writing–review and editing. **Parisa Najafi:** writing–original draft. **Jalil Hosseini:** writing–review and editing. **Mohammad Reza Shokouhamiri:** writing–review and editing. **Arezoo Asadi:** Supervision. **Roxana Parsapour:** Conceptualization.

## Ethics Statement

This study was approved by the Ethics Committee (code number: IR.SBMU.RETECH.REC.1402.378) of Shahid Beheshti University of Medical Sciences.

## Conflicts of Interest

The authors declare no conflicts of interest.

## Supporting information

Supporting information.

## Data Availability

All relevant data are included in the manuscript.
