# Supplementary material for: Acceptance‐Hesitancy of COVID‐19 Vaccination and Factors Affecting It in Adults: Systematic Review Study
Source: Immun Inflamm Dis. 2024 Nov 21;12(11):e70076. doi: 10.1002/iid3.70076 (PMC11580281; doi:10.1002/iid3.70076)
Supplement: Supplementary file 1 — Supporting information. [file IID3-12-e70076-s001.docx]

| **Table 1.** The descriptive statistics of the sample | | | | | | | | | | | | | | |
| --- | --- | --- | --- | --- | --- | --- | --- | --- | --- | --- | --- | --- | --- | --- |
| **No** | **Outcomes** | **Education**  **NO. %** | **Acceptance**  **%** | **Hesitancy**  **%** | **Refuse**  **%** | **Pregnancy** | **Dose** | **Type vaccine** | **No. of**  **Participants**  **Male%**  **(Mean age**  **± SD)** | **Participants**  **Characteristics** | **Study Design** | **Survey**  **modality** | **Country** | **Reference** |
| 1 | VH: side-effects (impact on fertility and pregnancy), Prior COVID diagnosis, vaccine effectiveness  VA: trust. | UD: 1745 (74.09)  NO UD: 645 (25.27) | 84 | 12 | 4 | NA | 1 | NA | 2491  25  NA | HCWs | CSS | Online | USA | (15) |
| 2 | VH: decreasing vaccine confidence, not being afraid of COVID-19, religious and cultural beliefs, mistrust and less confidence, financial loss | UD: 570 (56.6)  NO UD: 1029 (64.3) | 88 | NA | 11 | NA | 0 | NA | 1599  49  50 ± 16.23 | French-speakers | CSS | Online | Canada | (16) |
| 3 | VH: serious adverse effects, vaccine safety and efficacy, rapid development and implementation of vaccine, rapid development and implementation of vaccine | UD: 415 (100) | 63 | 35 | NA | NA | NA | mRNA  vaccine | 415  NA  NA | DS and MS | CSS | Online | USA | (17) |
| 4 | VH: short time of building vaccine, fear of vaccine needle, adverse effects, reinfection, type of vaccine, social media, losing family members | UD: 1021 (87.2)  NO UD: 150 (12.8) | 33 | 32 | 35 | NA | 0 | PFZ, AZ, Sinopharm | 1171  46  NA | Iraqi Kurdish population | CSS | Online | Iraq | (18) |
| 5 | VH: comments from TV, lack of health education about COVID-19, low income, and age  VA: health awareness and education | UD: 384 (80.2)  NO UD: 95 (19.8) | 40 | 29 | 30 | NA | 0 | NA | 479  45  NA | Residents of Sukkur City | CSS | Online | Pakistan | (19) |
| 6 | VH: serious side effects, lack of trust, and afraid of reinfection | UD: 810 (100) | 13 | 87 | 0 | NA | 1,2 | PFZ, AZ, Sinopharm | 810  40  NA | MS | CSS | Online | Iraq | (20) |
| 7 | VH: side effects, unsure of the vaccine, difficulties scheduling vaccination appointments | NA | 93 | 3.52 | 2.34 | NA | 1 | NA | 511  9  42.9 ±13.6 | HCWs | CSS | Online survey | USA | (21) |
| 8 | VH: side-effects, safety, COVID-19 is not severe enough, confidence in the vaccines, trusting public institutions | UD: 1813 (65.9)  NO UD: 938 (31.7) | 43 | 22 | 35 | NA | 0 | NA | 2753 51  NA | Hong Kong residents | CSS | Online | China | (6) |
| 9 | NA | UD: 218 (72.6)  NO UD: 82 (27.3) | 65 | 28 | 7 | NA | 0 | PFZ,Moderna,AZ,Janssen | 300  24  NA | Spanish citizens | CSS | Online | Spain | (22) |
| 10 | VH: safety, efficacy, and side effects, bad experience with a previous vaccination, having enough information | UD: 369 (36.5)  NO UD: 642 (63.5) | 69 | 31 | 0 | NA | 0 | PFZ, Moderna | 1011  45  46.9 ± 11.5 | Citizens | CSS | Online | Italy | (23) |
| 11 | VH: lack of trust in authorities, the vaccine approval process, the vaccines’ development velocity, health politics, and the pharmaceutical industry | UD: 4500 | 92 | 8 | 0 | NA | 1 | PFZ, Astra Zeneca | 4500  42  NA | HCWs | CSS | Online | Germany | (24) |
| 12 | VH: vaccine safety (long-term side effects). | 1450 (100) | 64 | 0 | 35 | NA | 2 | NA | 1450  35  46.3 ± 15.7 | CPs | CSS | Online | Italy | (25) |
| 13 | VH: safety, side effects, efficacy, and anti-vaccination beliefs and rumors | UD: 434 (56.7)  NO UD: 339 (43.3) | 31 | 69 | 0 | NA | NA | NA | 782  56  NA | Bangladeshi | CSS | Face-to-face and on-line | Bangladesh | (26) |
| 14 | VH: who had COVID-19 with severe symptoms, psychological difficulties, Lower levels of trust in media and health information sources and healthcare institutions, agreement with restrictions and higher levels of conspiracy mentality | UD: 487 (24.35)  NO UD: 1528 (76.4) | 65 | 28 | 7 | 0 | 1 | NA | 2,015  49  NA | Italian general population | CSS | Online | Italy | (27) |
| 15 | VH: trust CDC, vaccine safety, developed too quickly, | UD: 5252 (53.9)  NO UD: 4505(46.2) | 35 | 0 | 35 | NA | 1 | Johnson&Johnson | 54,727  58  NA | Adults | CSS | Online | USA | (13) |
| 16 | VH: safety, fear or dis(trust) in the authorities, or disinterest. | UD: 845 (85)  NO UD: 149 (14.9) | 36 | 26 | 38 | NA | 1 | NA | 994  NA  NA | PUB | CSS | By phone using CATI | Romania | (28) |
| 17 | VH: safety, side effects, effectiveness, effectiveness, religious beliefs, trust and rumor | UD: 65 (9.9)  NO UD: 503 (76.7) | 84 | - | NA | NA | 0 | NA | 655  56  35.13 SD:NA | Rural community | CSS | Face-to-face | Bangladesh | (29) |
| 18 | VH: fear of adverse reactions | NA | - | . | - | NA | 0 | NA | 18  Male: 8  NA | HCWs and CMs | CSS | IDI | India | (14) |
| 19 | VH: trust of government | UD: 2 940 (78)  NO UD: 828 (22) | Varicent groups | - | - | NA | 0 | NA | 3768  40  30± NA | MEXICAN Population | CSS | Online | Mexico | (30) |
| 20 | VH: difficulty in the vaccination request, registration protocols, negative social media reports, bad feelings, rumors, religious beliefs against, poor confidence, allergic reaction concerns, blood clot problems in women | UD: 1525 (100)  NO UD: 0 (0) | 29 | - | 71 | NA | 1,2,3 or more | NA | 1525  68  NA | HCW, Academics and Students | CSS | Online | Nigeria | (31) |
| 21 | VH: safety and efficacy, lack of information about the disease and vaccine or social media | UD: 507 (90.8)  NO UD: 51 (9.1) | 80 | 20 | 0 | NA | 1,2 | NA | 558  54  38.66 ±9.067 | Adults | CSS | Online | Saudi Arabia | (32) |
| 22 | VH: side effects and suboptimal efficacy, cost and the need for an annual booster, mistrust of medical personnel | NA | 56.2 | 20 | 19 | NA | 1 | NA | 2,564  90  NA | Jail residents | CSS | Door-to-door | USA | (33) |
| 23 | VH: vaccine’s side effects, long-term safety and efficacy, Vaccine will not be a solution for COVID | UD: 773 (37.7)  NO UD: 1278 (62.3) | 92 | 7 | NO | NA | 1 | NA | 2051  49  NA | General Population | CSS | Online and offline | India | (34) |
| 24 | VH: confidence and potential personal risk, insufficient evidence, side effects, long-term effects, speed of the vaccines’ development, general safety issues, general effectiveness and lack of knowledge | UD: 476 (100) | 94 | 4 | 1 | NA | 1 | NA | 476  37.5  21± 3.4 | University student | CSS | Online | UK | (35) |
| 25 | VH: side effects, getting coronavirus from the vaccine, efficacy, allergic to vaccines, mentions a conspiracy theory | - | 80.3% | - | - | - | 1 | - | 44,260  NA  NA | Adults in Lower-Middle-Income Countries | CSS | By phone | Africa, South Asia and Latin America, Russia, USA | (12) |
| 26 | VH: safety, rushed development, provide protection, reinfection | UD: 238 (81.2)  NO UD: 55 (18.8) | 63.54 | 27.42 | 9 | 299 | 1 | PFZ,Moderna,Janssen | 299  0  NA | Pregnant people | CSS | Live chat service | USA | (36) |
| 27 | VH: concern for brand and efficacy, side effects, safety | UD: 248 (100) | 88 | 12 | 0 | 7 | 1 | AZ, PFZ, Sino-Pharm, Sinovac | 248  64  NA | HCWs | CSS | In person or online platform | Pakistan | (37) |
| 28 | VH: too new, side effects, safety, efficacy, religious objections, not trust vaccines, role of politics in development. | UD: 13112 (75.7)  NO UD: 4186 (24.2) | 81 | 19 | 0 | NA | 1, 2 | PFZ-BioNTech, Moderna, AZ | 21,294  23  NA | Cancer, Autoimmune Diseases | CSS | Online | USA | (38) |
| 29 | VH: side effects, safety | UD: 408 (45.0)  NO UD:498 (55.0) | 69 | 31 | 0 | NA | 1 | NA | 906  39  NA | PwMDs | CSS | Online or paper-pencil manner | China | (39) |
| 30 | VH: side effects, Vaccine needs more research, expedited vaccine trials, incomplete information, recommendation by the government, lack of information about the vaccine | UD: 3526  NO UD: 247 | 54 | 23 | 13 | NA | 1 | NA | 3773  27  NA | Students | CSS | Email | USA | (40) |
| 31 | VH: trusted the efficacy and safety. Trust in government and concerns about the impact of vaccine on disease | NO UD: 97 (51.9)  UD: 88 (47.1)  Not acquired: 2 (1.1) | 46 | 54 | 0 | NA | 1 | NA | 187  NA  64.2± 9.2 | PD | CSS | Online | China | (41) |
| 32 | VH: trust vaccine, illness concern, safety, Trusting vaccine safety | NA | 78 | 0 | 22 | NA | 1 | NA | 168  69  NA | Pennsylvania Autism | CSS | Email | USA | (42) |
| 33 | VH: adverse events, efficacy, vaccination fee | UD: 5092 (71)  NO UD: 2118 (29) | 48 | 18 | 34 | NA | 0 | NA | 7210  47  NA | General population and HCWs | CSS | Online | Japan | (43) |
| 34 | VH: adverse effects, lack of information, afraid of the queue | UD: 185 (60.3)  NO UD: 122 (39.8) | 94 | 6 | 0 | NA | 1 | NA | 307  56  24.15± 6.8 | Population | CSS | Online  (social media) | Nepal | (44) |
| 35 | VH: side effects, safety, getting COVID-19 from the vaccine, cost | UD: 3938 (85.1)  NO UD: 690 (14.9) | 63 | 37 | 0 | NA | 0 | NA | 4630  33  41.63 ± SD | HCWs | CSS | Google Forms | Iran  multicenter | (45) |
| 36 | VH: trust, efficacy, safety, painful administration, effectiveness | UD: 205 (50)  NO UD: 205 (50) | 53 | 47 | 0 | NA | 0 | NA | 410  43  18-51+ | Population | CSS | Face to face | Pakistan | (46) |
| 37 | VH: infected by COVID-19, efficacy, side effects, trust pharmaceutical companies and public authority, how the vaccine works, using barrier gestures, media and social network | UD: 3089 (100.) | 58 | 25 | 17 | NA | 0 | NA | 3089  30  20.3±1.9 | Students | CSS | Online | France | (47) |
| 38 | VH: side effects, unsafe, not useful, effectiveness, social media, immune system, religious reasons, unnecessary, Prior exposure, Prior chronic conditions | UD: 343 (26)  NO UD:974 (74.0) | 60 | 0 | 40 | NA | 1,2 | AZ | 1325  47  51.1± 9.35 | VI and non-VI | CSS | Telephonic interviews | Pakistan | (48) |
| 39 | VH: lack of information, people get the vaccine first, needles/injections, not have enough time to take decision, trust the experts ND, risks of the vaccine, efficacy, COVID-19 is not dangerous | UD: 2,761 (100) | 81 | 14 | 5 | NA | 0 | PFZ | 2,761  28  NA | HCWs | CSS | Email | Canada | (49) |
| 40 | VH: side effects, safety and effectiveness, lack of hindsight, short time of production. | UD: 200 (100) | 35.5 | 57.5 | 6.5 | NA | 1 | PFZ,Moderna | 200  46  NA | French-speaking students | CSS | Interview | France | (50) |
| 41 | VH: safety and side effects | UD: 918 (57.4)  NO UD: 1094 (42.6) | 81 | 19 | 0 | NA | 1 | Sinovac,PFZ | 2,012  2  39±8.1 | Ethnic minorities | CSS | Online | China | (51) |
| 42 | VH: risks and safety, confidence, complacency, constraint, calculation, collective responsibility | 528 (100) | 95 | 5 | 0 | Exclude | 1, 2 | PFZ | 528  12  NA | HCWs | CSS | Online | Singapore | (52) |
| 43 | VH: efficacy, safety | NO UD: 8823 (84.3)  UD: 1973 (21.4) | 62 | 9 | 28 | NA | 0 | NA | 10796  42  NA | Mexican population | CSS | NA | Mexico | (53) |
| 44 | VH: trust in institutions and public health | UD: 3995 (53)  NO UD: 3610 (47) | 82 | 18 | 0 | NA | 0 | NA | 7605  34  NA | HCPs  And GDP | CSS | Online (social platforms ( | Italy | (54) |
| 45 | VH: misinformation and fear, concerns about safety, lack of information, trust issues | UD: 736 (84.5)  NO UD: 135 (15.5) | 88 | 0 | 12 | NA | 0 | NA | 871  47  NA | Egyptians | CSS | Online | Egypt | (55) |
| 46 | VH: safety and effectiveness, negative information, rushed development, vaccine brand, fear of needles | UD: 217 (100) | 56 | 44 | 0 | 0 | 0 | AZ | 217  46  NA | MS | CSS | Online (social media) | Sudan | (56) |
| 47 | VH: trust in the healthcare system, social media disinformation, Safety, | UD: 4958 (72.0)  NO UD: 984 (14.0) | 69 | 0 | 31 | NA | 0 | NA | 6883  46  NA | Social media users | CSS | Online | USA | (57) |
| 48 | VH: safety and efficacy | 419 (100) | 69 | 21 | 10 | NA | 0 | NA | 419  21  NA | HCWs | CSS | In-person and online | Poland | (58) |
| 49 | VH: baby's health, trust and confidence in vaccine safety | NO UD: 253\253: (100)  UD: 0 (0) | 59 | 25 | 14 | 287 | 0 | NA | 287  0  32.8 ±5.1 | Pregnant  women | CSS | Online | Australia | (59) |
| 50 | VH: fear, religious reasons, lack of trust, safety, not enough information. | UD: 490 (79.3)  NO UD: 128 (20.6) | 44 | 23 | 33 | NA | 0 | NA | 618  49  NA | Adult | CSS | Online | Nigeria | (60) |
| 51 | VH: efficacy, adverse events, social, degree of self-perceived vaccine literacy. | 705 (100) | 96 | 4 | 0 | NA | 0 | Na | 705  49  NA | Physicians | CSS | Online | Thailand | (61) |
| 52 | VH: medical mistrust, structural barriers, safety and efficacy | UD: 389 (52)  NO UD: 353 (48) | 60 | NA | 40 | NA | NA | NA | 730  51  NA | Americans | CSS | Online | USA | (62) |
| 53 | VH: adverse side effects, safety efficacy, short duration of clinical trials, vaccine approve mechanisms. | 5312 (100.0) | 59 | 0 | 41 | NA | 1 | Sinovac,AZ, PFZ, Moderna, Cocktail | 5312  16  NA | VHV | CSS | Online | Thailand | (63) |
| 54 | VH: Side effects, health, concerns and newness, safety and efficacy, trust | UD: 467 (59.7)  NO UD: 315 (40.3) | 82 | 9 | 5 | NA | 0 | Johnson & Johnson, PFZ, Moderna | 789  19  NA | HCWs | CSS | Online | USA | (64) |
| 55 | VH: side effects, short period of vaccine development, lack of trust and information, had COVID-19, after most people take, building immunity through COVID-19 infection | UD: 434 (82)  NO UD: 97 (18) | 62 | 38 | 0 | NA | 0 | NA | 531  60  NA | Adults | CSS | Online | Saudi Arabia | (65) |
| 56 | VH: fear of adverse effects, lack of vaccine confidence, Safety | 631 (100) | 78 | 11 | 11 | NA | 1 | NA | 631  20  20.08 ± 1.7 | Health Care Students | CSS | Online | China | (66) |
| 57 | VH: lack of belief in vaccination, efficacy, waiting for a better vaccine, adverse events. | UD: 408 (75)  NO UD: 133 (25) | 58 | 42 | 0 | NA | 1 | Sinopharm, Sinovac, AZ | 541  50  NA | General population | CSS | Interview | Pakistan | (67) |
| 58 | VH: side effects, trust, rushed development, efficacy, will not stop the infection, consume herbal concoction to Prevent COVID-19, don’t need vaccine | UD: 511 (89)  NO UD: 61 (11) | 13 | 0 | 87 | NA | 0 | NA | 572  23  NA | Cameroonians | CSS | In-person and online | USA | (68) |
| 59 | VH: side effects, trust, exaggerated virus impact | UD: 512 (38.79)  NO UD: 761 (61.26) | 71 | 15 | 14 | NA | 0 |  | 1284  57  NA | New Zealanders | CSS | Online | New Zealand | (69) |

**VH**: Vaccine Hesitancy, **VA**: Vaccine Acceptance, **UD**: University Degree, **No UD**: No University Degree, **HCW**: Healthcare workers, **CSS**: Cross-Sectional Stud, **DS**: Dental Students, **MS**: Medical Students, **PFZ**: Pfizer, **AZ**: AstraZeneca, **MS**: Medical Students, **CPs**: Community Pharmacists, **PUB**: Public, **CMs**: Community members, **IDI**: In-depth Interview, **PWMDs**: Persons with mental disorders, **PD**: Parkinson’s Disease, **VI**: Vaccinated Individuals, **Non VI**: Non- Vaccinated Individuals, **HCPs**: HealthCare Professional, **GDP**: General Adult Population, **MS**: Medical student, **VHV**s: Village Health Volunteer.
